# Indicated school-based intervention to improve depressive symptoms among at risk Chilean adolescents: a randomized controlled trial

**DOI:** 10.1186/s12888-016-0985-4

**Published:** 2016-08-04

**Authors:** Jorge Gaete, Vania Martinez, Rosemarie Fritsch, Graciela Rojas, Alan A. Montgomery, Ricardo Araya

**Affiliations:** 1 Centre for Global Mental Health, Department of Population Health, London School of Hygiene and Tropical Medicine, Keppel Street, London, WC1E 7HT UK; 2 Departamento de Salud Pública y Epidemiología, Facultad de Medicina, Universidad de los Andes, Monseñor Álvaro del Portillo 12455, Las Condes, Santiago Chile; 3 Millennium Institute for Research in Depression and Personality, Av. Vicuña Mackenna 4860, Macul, Santiago Chile; 4 Universidad de Chile, Centro de Medicina Reproductiva y Desarrollo Integral del Adolescente, Facultad de Medicina, Universidad de Chile, Av. Profesor Zañartu 1030, Independencia, Santiago Chile; 5 Universidad de Chile, Departamento de Psiquiatría y Salud Mental, Clínica Psiquiátrica Universitaria, Av. La Paz 1003, Recoleta, Santiago Chile; 6 Nottingham Clinical Trials Unit, University of Nottingham, Nottingham, UK

**Keywords:** Indicated-school based intervention, Depression, Adolescents, Prevention

## Abstract

**Background:**

Depression is a disabling condition affecting people of all ages, but generally starting during adolescence. Schools seem to be an excellent setting where preventive interventions may be delivered. This study aimed to test the effectiveness of an indicated school-based intervention to reduce depressive symptoms among at-risk adolescents from low-income families.

**Methods:**

A two-arm, parallel, randomized controlled trial was conducted in 11 secondary schools in vulnerable socioeconomic areas in Santiago, Chile. High-risk students in year 10 (2° Medio) were invited to a baseline assessment (*n* = 1048). Those who scored ≥10 (boys) and ≥15 (girls) in the BDI-II were invited to the trial (*n* = 376). A total of 342 students consented and were randomly allocated into an intervention or a control arm in a ratio of 2:1. The intervention consisted of 8 group sessions of 45 min each, based on cognitive-behavioural models and delivered by two trained psychologists in the schools. Primary (BDI-II) and secondary outcomes (measures of anxiety, automatic thoughts and problem-solving skills) were administered before and at 3 months post intervention. The primary outcome was the recovery rate, defined as the proportion of participants who scored in the BDI-II <10 (among boys) and <15 (among girls) at 3 months after completing the intervention.

**Results:**

There were 229 participants in the intervention group and 113 in the control group. At 3-month follow-up 81.4 % in the intervention and 81.7 % in the control group provided outcome data. The recovery rate was 10 % higher in the intervention (50.3 %) than in the control (40.2 %) group; with an adjusted OR = 1.62 (95 % CI: 0.95 to 2.77) (*p* = 0.08). No difference between groups was found in any of the secondary outcomes. Secondary analyses revealed an interaction between group and baseline BDI-II score.

**Conclusions:**

We found no clear evidence of the effectiveness of a brief, indicated school-based intervention based on cognitive-behavioural models on reducing depressive symptoms among Chilean adolescents from low-income families. More research is needed in order to find better solutions to prevent depression among adolescents.

**Trial registration:**

Current Controlled Trials ISRCTN33871591. Retrospectively registered 29 June 2011.

**Electronic supplementary material:**

The online version of this article (doi:10.1186/s12888-016-0985-4) contains supplementary material, which is available to authorized users.

## Background

Depression is common worldwide and starts at an early age. Among adults diagnosed with major depressive disorder at age 26, 51.3 % had been first diagnosed between ages 11 and 15 [1]. Several studies have found a 12-month prevalence of depression around 4 to 5 % among adolescents [2, 3]. In Chile, the 12-month prevalence rate of major depressive disorder among adolescents has been estimated as 6.9 % [4]. Depression is a major risk factor for suicidal behaviour [5, 6], is associated with serious social and academic impairments [7], and increases the risk of other health problems [8], substance misuse [9] and obesity [10] later in life.

Several preventive and treatment initiatives for depression among adolescents have been tested, especially using Cognitive behavioural therapy (CBT) approaches, since it has been found effective to improve depressive symptoms among children and adolescents [11].

Preventive interventions may be classified into three types [12]: i) Universal interventions delivered to the whole population; ii) Selective interventions targeted to a subgroup of the population whose risk of developing an emotional or behavioural problem is higher than the average; and iii) Indicated interventions targeting individuals who already have signs or symptoms of an emotional or behavioural disorder. Thus, selective and indicated prevention interventions can be considered “targeted” preventive approaches [12]. Cognitive behavioural interventions have been evaluated for a long time and there are some successful examples for universal [13] and targeted interventions [14, 15].

Most school-based interventions have used universal rather than targeted approaches [16–20]. Universal programs have shown small effects [16, 21] but targeted interventions have yield better results [16, 20, 22]. It has been postulated that targeted interventions are more effective because groups are smaller, often students present more depressive symptoms at the start and may be more receptive to the content of these interventions, and there is more room for improvement since baseline depression scores are higher [16, 20]. Additionally, the social impact would probably be more noticeable as these adolescents often require immediate help.

A meta-analysis found that effectiveness was larger among programs targeting high-risk individuals using cognitive-behavioural approaches, and when professionals delivered the programmes [21]. However, other review found less promising results for indicated interventions [19], suggesting the need for more research in this field.

In a recent report, we presented the results of the first large randomized controlled trial of a school-based universal intervention to decrease depressive symptoms among adolescents in Chile [23]. This 11-session intervention was based on a cognitive-behavioural therapeutic approach with two main components: cognitive re-structuring (six sessions) and teaching problem-solving strategies (three sessions). It also included booster sessions, and the facilitators were not necessarily health or psychology professionals. We did not find any clinically important differences between intervention and control groups at follow-up.

We took into account the experience gathered in the previous universal school-based trial, reviewed the intervention, and decided to introduce some changes, to make it shorter and sharper, and increased the interaction between participants and facilitators. Firstly, we decided to focus on adolescents who already have some symptoms. Secondly, we reduced the number of sessions. We detected some redundancy in the content and duration of the original intervention, in particular in the cognitive re-structuring section. Therefore, we condensed this section into 3 sessions, reducing to 8 the total number of sessions. There is large variability in the duration or number of sessions of previously tested interventions. This is important because there is some evidence that the number of sessions may influence the effectiveness of an intervention. In a recent review [20], 22 out of 25 targeted interventions studies provided information about the number of sessions, ranging from 4 to 36 (mean 10.5: *SD* = 6.7). Other reviews found that interventions with 8 to 12 sessions achieved better results than those with more or fewer sessions [16] and that a minimum of 6 sessions is required to obtain changes [24]. However it is yet unclear what is the ‘ideal’ minimum number of sessions and whether this can influence the magnitude and duration of any improvements. Thirdly, we introduced more interactive activities such as role-playing. And finally, we used well-trained mental health professionals as facilitators to deliver the interventions.

The objectives of the study reported here were to determine the effectiveness of an 8-session, indicated, school-based intervention using well-trained professionals to reduce depressive symptoms among Chilean adolescents and to improve functioning, as well as to assess the role of mediating factors such as newly acquired problem solving skills and reduction in dysfunctional negative thoughts.

Hypotheses:

1. There will be an absolute difference of 20 % in recovery rate between intervention and control groups at 3 months after completing the intervention.
2. Adolescents in the intervention group will show improved levels of functioning.
3. Adolescents receiving the intervention will show greater reductions in negative thoughts and improvements in problem solving skills than those in the control group.

## Methods

### Trial design

This was an individually randomised controlled trial. We randomised students with a 2:1 allocation ratio, a decision based on the need to reduce costs [25].

### Settings, participants, and eligibility criteria

At the time of the study, municipal schools provided education to almost half of all secondary school students in Santiago, with virtually all of them coming from low socio-economic families. All students attending “2° Medio” grade (equivalent to 10 years of education) from eleven municipal schools taking part as control schools in the previous study [23] were invited to participate. Informed consent was obtained from parents or main caregivers and assent from the students.

According to Beck at al. (5), adolescents with BDI > 14 could be considered as having a clinical depression. However in our recent study we found that the best cut-off point differs between boys and girls; for instance, the optimal cut-off point was 19/20 for girls (sensitivity 74,5 % and specificity 73,8 %) and 13/14 for boys (sensitivity 72.2 % and specificity 64.1 %) [26]. Nonetheless, at the time of planning this study we did not have this information, so we decided to use a differential optimal cut-off point for boys and girls based on other dataset. As such we used cut-off points of 9/10 for boys and 14/15 for girls. Using these cut-off points with baseline data from our universal school-based intervention mentioned above [23], we expected that 40 % of adolescents would be eligible to enter this study. Therefore, the eligibility criteria were: i) adolescents attending 2° Medio in a municipal school participating as control schools in our previous study [23], and ii) having a BDI score ≥10 (among boys) and ≥15 (among girls).

### Randomization

Randomization was stratified by school. Allocation to groups was concealed and took place after all students were recruited in each school. A list of all students who provided consent was generated for each school. An independent statistician, using a computer-generated list of random numbers, allocated students to intervention and control groups in each school using a ratio of 2:1. After individuals were randomly allocated to arms, an independent person formed the intervention groups within the active arm trying to maintain a reasonable balance by sex.

### Intervention

The intervention was a modified version of the CBT-based program YPSA - I (*Yo*), Think (*Pienso*), Feel (*Siento*), Act (*Actuo*) used in our school-based universal intervention [23]. The main modifications were: the number of sessions was reduced from 11 to 8, some sessions were improved according to feedback received in the previous trial, and trained psychologists delivered the intervention.

The revised program (YPSA-R) consisted of 8 weekly sessions each lasting 45 min. There was an introductory session, three sessions dealing with thought re-structuring, three sessions on problem solving skills and one closing session with a revision of the previous learning and planning for the future. Two trained psychologists (facilitators) for each group delivered the intervention. If more than one group took place in a given school, the same facilitators delivered the intervention for all groups in that school, for practical and logistical reasons. Facilitators had a detailed manual specifying key learning points and objectives for each session and received 2 days of training that covered the identification and management of mental health problems, group management techniques as well as training to deliver the specific intervention. The intervention was fully manualized. During the course weekly supervision groups were provided for facilitators during which fidelity checks were performed. A supervisor met with facilitators and checked if content and methods were used and delivered as intended. The supervisor was an experienced senior clinician from the local team. This supervisor participated in the previous trial so that she was familiar and knowledgeable about the intervention. One of the lead authors was available to offer support and advice to the supervisor in logistical issues when needed. The size of each of the intervention groups was between 8 and 15, trying to achieve a balance in sex ratios in each group. Considering that there were two facilitators per group, the size of the group was manageable and allowed for a more personalized intervention.

Students in the intervention arm were contacted prior to the first session to explain the procedure to follow for conducting the sessions. Essentially students were told the time and place where the sessions would be delivered. Head teachers were informed of this so that students were given permission to be absent from some classes if this was needed. No explanation was given to other students for this absence. Whenever possible, sessions were delivered after school time.

### Control group

The control group received nothing other than the normal teaching activities and assessments.

### Outcomes measures

#### Primary outcome

Beck Depression Inventory II (BDI-II) [27] was used to assess depressive symptoms and to determine recovery rate. This is a brief and well-established depression questionnaire translated to different languages and used widely throughout the world. It has previously been used among adolescents in Chile and in other Latin-American countries showing good psychometric properties [26, 28]. The internal consistency of this instrument was 0.82. It is self-completed which has the advantage of reducing potential observer bias since it is unlikely that observers will be completely blind to allocation. The BDI-II also provides a good measure of the cognitive changes expected to occur with the intervention. For the primary analysis, the recovery rate was defined as the proportion of students with BDI-II score <10 for boys or <15 for girls, three months after the intervention was completed. For secondary analysis, this variable was treated as a continuous measure.

#### Secondary outcomes

Revised Child Anxiety and Depression Scale (RCADS): this is an adaptation of the Spence Child Anxiety Scale (SCAS) [29] and intends to assess symptoms of DSM-defined anxiety disorders and major depression. The scale consists of 47 items that on the basis of exploratory factor analysis are allocated to six subscales: Social phobia (9 items); Panic disorder (9 items); Major depressive disorder (10 items); Separation anxiety disorder (7 items); Generalized anxiety disorder (6 items); and Obsessive-compulsive disorder (6 items). Items have to be scored on a 4-point scale. RCADS subscale scores can be obtained by summing across relevant items. We excluded the depression, separation anxiety, and obsessive-compulsive sub-scales because depression was already assessed using the BDI-II and separation anxiety and obsessive-compulsive disorder were regarded as less prevalent for this age. The internal consistency of the 15 items included in this study was 0.79.

#### Measures of psychological functioning

Children’s Automatic Thoughts Scale (CATS) [30]: This self-completed scale assesses a range of negative self-statements in children and young people aged 7–16. For each item the child is asked to rate whether they have had a similar thought over the past week. Each item is rated as “not at all” (scores 0), “sometimes” (scores 1), “fairly often” (scores 2), “often” (scores 3) or “all the time” (scores 4). Confirmatory factor analysis identified 4 distinct but correlated factors relating to thoughts about physical threat, social threat, personal failure and hostility. Internal consistency for the total score was high (Cronbach Alpha = 0.95) with acceptable test–retest reliability (0.79). The scale has been found to effectively discriminate between a community and clinical sample with the personal failure sub-scale being the strongest predictor of depressive symptoms. The 10-item personal failure was the only sub-scale used because it was the most useful to detect negative thinking something we were aiming to change with the intervention [31] and its internal consistency was 0.87.

The Short Form of the Social Problem-Solving Inventory Revised (SPSI-R Short Form) [32] was used to assess problem-solving dimensions. The original SPSI-R Short Form consisted of 25-item self-report instrument that measures two adaptive problem-solving dimensions (positive problem orientation and rational problem solving) and three dysfunctional dimensions (negative problem orientation, impulsivity/carelessness style, and avoidance style). Each item is rated on a 5-point scale ranging from not at all true of me (0) to extremely true of me (4). However, an exploratory factor analysis of the data from pre-study pilot showed that 5 items did not loaded in the one-factor solution; therefore, they were removed from the questionnaires self-completed by students in this study, ending with a scale of 20 items, with an internal consistency of 0.92. No sub-scales will be used in the analysis.

Other variables measured were age, sex, alcohol and cannabis use.

### Sample size

We aimed to find an effect size of 20 % in recovery rates between intervention (60 % recovered) and control group (40 % recovered). An individually randomized trial with 2 arms, in a ratio of allocation of 2:1 would require approximately 200 individuals in the intervention arm, and around 100 individuals in the control arm. This size would allow detecting this absolute difference with 81 % power and a 2-sided 5 % significance level. In any case, there would be enough power to test a difference of 3 points (between 22 and 19 points, equivalent to 0.3 SD) across arms in the BDI-II scores with a 5 % alpha and 80 % power, as part of our secondary analysis. We considered this difference to be clinically significant.

### Statistical analyses

Descriptive statistics were used to assess balance across arms at baseline. The primary between-group analysis was carried out on an intention-to-treat basis for 3-month BDI-II scores representing proportions of students recovered using logistic regression analysis, unadjusted and adjusting for baseline BDI-II score, age and sex. We also investigated any evidence of clustering of depressive symptoms by schools [26]; however, no marked clustering effect was found (results available on request).

Secondary analyses comprised BDI-II as a continuous measure using a regression analysis adjusting for baseline BDI-II score, age and sex. Also the means of all secondary outcome measures (RCADS, CATS and SPSI-R Short Form) were compared across groups using a similar analytical strategy as above.

We conducted pre-planned subgroup analyses for the primary outcome using interaction terms in the regression models for sex and BDI-II scores at baseline.

Sensitivity analyses were conducted to assess the effects of missing data using multiple imputations. Results with and without imputed data were similar, therefore we only present complete data analysis results (imputed data results available upon request).

We also performed a Complier Average Causal Effect (CACE) analysis to assess the impact of the number of sessions on the main outcome. We defined a priori compliance as attendance to at least 7 sessions as we hypothesised that this would deliver enough components of the intervention. This decision was mainly based on others studies which considered no less than 6 sessions [24] or close to 8 [20], as good ‘doses’ of similar interventions.

All analyses were performed using STATA 12.01.

## Results

**Fig. 1:**
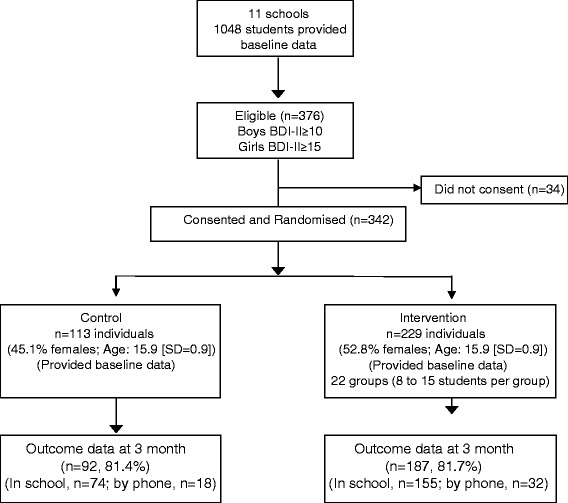
Consort flow diagram

A total of 1048 students participated in the control arm at 12-month follow-up of the original study. Nearly 36 % of them (*n* = 376) were eligible for this study as they had BDI-II scores above cut-off points defined previously. All but 34 students consented to participate and were randomly allocated in a proportion of 2:1 to the intervention (*n* = 229) and control group (*n* = 113). Primary outcomes at 3 months were available for 81.7 % (*n* = 187) in the intervention group and for 81.4 % (*n* = 92) in the control group. Figure 1 shows the flow of students in the study.

Overall trial arms were reasonably well balanced in terms of age, clinical measures, binge drinking and cannabis use. However, the intervention group had more women than the control group (See Table 1). The mean age for the whole sample was 15.9 with a 73.9 % between 15 and 16 (age ranged between 14 and 19).

**Table 1:** Characteristics of participants at baseline by trial arm

		Trial Arm	
Variable	Level	Control *n* = 113	Intervention *n* = 229
Sex	Male	62 (54.9)	108 (47.2)
	Female	51 (45.1)	121 (52.8)
Mean Age (SD)	Years	15.9 (0.9)	15.92 (0.9)
Mean BDI II score (SD)		21.9 (8.5)	22.53 (9.3)
Mean CATS score (SD)		14.1 (9.0)	15.46 (9.2)
Mean RCADS score (SD)		24.0 (7.7)	24.07 (8.8)
Binge drinking (last 30 days)	Never	73 (65.2)	147 (64.2)
	1-2 times	21 (18.8)	46 (20.1)
	3 or more times	18 (16.1)	36 (15.7)
Cannabis use (last 30 days)	Yes	28 (24.8)	54 (23.6)

Figures are percentages unless otherwise stated. Binge drinking: 5 or more drinks in one occasion

The 229 students in the intervention arm were arranged into 22 groups with a median of 10 (IQR = 3) students per group and a range between 8 and 15 per group.

The mean number of sessions attended by each student was 4.4 (SD = 2.4; median = 5.0; IQR = 3.0; range, 0–8 sessions), with 25.2 % of students attending at least 7 sessions. The average attendance rate per session was 55.5 % of the participants (SD = 5.9; range, 45.0–66.4 %)

At three months post-intervention, the recovery rate in the intervention group was approximately 10 % higher than in the control group (recovery rate in control group = 40.2 %: Recovery rate in intervention group = 50.3 %; *p* = 0.08) (see Table 2).See also "Annex: Primary analysis with imputed data: recovery rate at 3 months" in the Additional file 1, with the results with imputed data.

**Table 2:** Primary analysis with observed data: recovery rate at 3 months

Control (*N* = 92) *n* (% recovery rate)	Intervention (*N* = 187) *n* (% recovery rate)	OR (95 % CI)	*p*-value
		Unadjusted	
37 (40.2)	94 (50.3)	1.50 (0.91 to 2.49)	0.115
		Adjusted^a^	
		1.62 (0.95 to 2.77)	0.08

^a^The adjusted model included sex, age and baseline BDI-II. Recovery rate refers to proportion of participants who scored <10 (among boys) and <15 (among girls) in the BDI-II at 3 months after completing the intervention

There was no evidence of an interaction between group and sex (Ratio of Odds Ratios [ROR] = 1.08; 95 % CI:0.39 to 2.98; *p* = 0.883); however, there was an interaction between group and baseline BDI-II score, in the intervention group (ROR = 0.93; 95 % CI: 0.87 to 0.99; *p* = 0.016).

There was some variation in recovery rates among students across schools in the intervention arm (from 40.91 % to 83.33 %; difference 42.42 %) but also in the control arm (from 22.22 % to 66.67 %; difference 44.45 %).

The secondary analysis showed no difference between intervention and control group in BDI-II mean scores at 3 months follow-up before and after controlling by sex, age and baseline BDI-II (See Table 3).

**Table 3:** Secondary analysis with observed data: BDI-II mean scores at 3 months (Unadjusted and Adjusted for sex, age & baseline BDI-II)

Control (*N* = 92) Mean (95 % CI)	Intervention (*N* = 187) Mean (95 % CI)	β (95 % CI)	*p*-value
		Unadjusted	
15.2 (13.1 to 17.3)	15.1 (13.6 to 16.6)	−0.16 (−2.76 to 2.43)	0.901
		Adjusted^a^	
		−0.76 (−3.21 to 1.69)	0.544

^a^The adjusted model included sex, age and baseline BDI-II

Regarding the CACE analysis, it showed no evidence of any intervention effect with the estimated difference between intervention and control arm of −0.14 (−2.38 to 2.10; *p* = 0.901). Assessing the additional intervention effect per session attended gave an estimated difference of −0.04 (−0.60 to 0.53; *p* = 0.901).

Regarding secondary outcomes, we found no difference between control and intervention groups (See Table 4).

**Table 4:** Secondary outcomes and psychological measures at 3 months

Variable	Control mean (95 % CI)	Intervention mean (95 % CI)	β (95 % CI)^a^	*p*-value
RCADS scores	20.9 (19.3 to 22.5)	20.3 (19.0 to 21.7)	−1.2 (−3.1 to 0.8)	0.234
CATS scores	11.2 (9.4 to 13.1)	11.3 (10.0 to 12.5)	−0.9 (−2.6 to 1.1)	0.438
SPSI-R SF scores	42.2 (39.1 to 45.3)	42.7 (40.2 to 45.1)	0.8 (−3.0 to 4.6)	0.666

^a^This was an adjusted model including sex, age and baseline measures

## Discussion

As far as we know this is the first randomised controlled trial in Latin America of an indicated school-based intervention aiming to reduce depressive symptoms among adolescents. In a previous study we tested a universal but similar school-based intervention and found that it was not more effective than a control group. Thus, on this study we tested a similar intervention but using a targeted approach focusing only on those students who presented depressive symptoms at baseline. Trained professionals delivered the intervention to smaller groups. As in the previous trial, we found no evidence of increased effectiveness of this targeted intervention compared to the control group using complete data and multiple imputation analyses.

Notwithstanding these negative results, the recovery rate in the intervention group was almost 10 % higher than in the control group but it did not reach the statistical significance we had envisaged. It is possible that there is a small difference in recovery rates across groups but our study did not have enough statistical power to test a smaller difference as the one observed. If we convert this absolute risk reduction (10 %) into the number needed to treat (NNT), we obtain a NNT of 10, in other words, 10 students with similar characteristics of those included in this study, need to be treated in order for one to benefit with this intervention. This is considered as a moderate effect size [33, 34]. Whether or not a difference of 10 % across arms would justify the implementation of an intervention such as the one tested here it is something that would need to be explored further.

Comparatively, a recent study in the US [35] showed good results using a similar cognitive behavioural approach. As in our trial, this US study used cognitive restructuring (challenging and replacing automatic negative thoughts) as a key component of the intervention and delivered the intervention in small groups with lower number of sessions. However, these two interventions differed in several aspects. Even though both studies included training in problem solving skills and emphasis on understanding the cognitive-behavioural model (e.g., how the feelings affect the way we think and behave), the US intervention included motivational messages to get the students involved in pleasurable activities. In our study the groups were mixed and the facilitators were always a couple of trained young psychologists whilst in the US study groups were of same sex and facilitators were school staff. Finally, even though the number of sessions offered in our study was higher (8 instead of 6), attendance to our intervention sessions was lower (mean session attended: 4.4 vs. 5.3) perhaps reflecting the impact of motivational messages in the US study. We could not compare the participants in both studies regarding substance use and socioeconomic status.

There are very few studies of the effectiveness of psychological interventions from low-middle income countries to be able to compare. However, we have conducted other group interventions in Chile for other population sub-groups showing that attendance is and remains a challenge. For instance, in our study with depressed women in primary care the attendance was around 6 out of 9 sessions [36], and in our post-natal depression trial it was 3 out of 8 sessions [37]. We continue to explore how best to improve this including more agile and shorter sessions, involving other people to support compliance, and using other means for delivering interventions such as mobile devices or social media.

Among the main reasons we have considered to explain the negative results of our trial is the fact we had a lower than expected attendance to the sessions. Many factors may have contributed to this: i) it is known that adolescents with depressive symptoms (lack of energy and reduced motivation) have higher rates of school absenteeism [38]; ii) participation and attendance to sessions was voluntary and we provided no incentives to attend the sessions; iii) on most occasions the sessions took place after school hours and participants might have had more attractive activities to do; and iv) there was no involvement of parents so we could not rely on their help and support to encourage their children to attend sessions. Unfortunately, we do not have data regarding the general school attendance of the participants to explore if the low attendance to sessions was correlated with school absenteeism. Regarding incentives, Rohde et al. found positive results with a 6-sesssion intervention with a mean attendance of 5.3 sessions. It is worth mentioning that this intervention included incentives such as snacks for all and gift cards for participants who attended 6 sessions [24]. In our study, the Ethics Committee did not allow the use of any kind of incentives for participation. As far as parental support is concerned, a successful study [15] that included parental support reported a mean number of sessions attended of 6.5 out of 8. Another successful study [14] testing a 15-session cognitive intervention reported a mean number of sessions attended of 9.5, but also included an invitation to three parental meetings. These are some possibilities to be explored in future research to improve compliance with psychological interventions among adolescents.

Another potential reason may be that the content of the intervention did not cover the wide range of needs of these participants. These students with depressive symptoms may also have other problems, such as substance abuse or bullying [9, 39–43]. If these other problems, for instance substance abuse, were the primary cause of depressive symptoms, an intervention mostly focused on depressive symptoms rather than on substance use might not be sufficient to improve the condition of these adolescents. A recent systematic review identified five studies testing indicated interventions [19]. The results of these studies were mixed, three achieved positive results but two failed to do so. Interestingly, among those studies with positive results the interventions included content not exclusively related to depression and the primary outcomes also included personal control and suicidal behaviour [44, 45], and substance misuse [45, 46]. By contrast, the interventions with negative results were more restricted to depressive symptoms using cognitive re-structuring and developing strategies of coping and problem solving skills [47, 48]. However, a more recent study using a broad and brief, school-based selective preventive intervention aiming to reduce substance use and mental health problems among adolescents failed to find positive effects up to 12 months post-intervention follow-up [49]. For instance, we found that one in four students used cannabis in the last 30 days (prevalence higher that the general population of similar age in Chile) [50], and one third had consumed 5 or more drinks at least one day in the last 30 days. So, this is a population not just with emotional symptoms but also with risky substance use behaviours, a problem that the intervention did not address. Furthermore, students in our study attended schools in areas with marked social deprivation and socio-economic difficulties. Nonetheless we tried to address this potential barrier through introducing problem solving sessions but it seems that the students were not able to improve on these skills as shown in the secondary outcomes analysis.

Additionally, we may need to consider that the procedures used to deliver the intervention (setting, format and rapport with facilitators) may not be very appealing to these students. Even though, the intervention appears to have attractive features for general adolescents (data not shown and collected in previous study) [23], there are important changes on how young people communicate with each other, using mobiles and social media [51], and this intervention used a more traditional approach, something that it might be less engaging to them.

There is some evidence of a moderation effect of therapists on the efficacy of different psychosocial interventions [52]. It is for this reason that we decided that all facilitators in our intervention would be clinical psychologists, trained and well supervised during the whole study by an experienced psychologist. Each school had assigned two facilitators working as a team. There was some variation in outcomes across schools in the intervention arm but there was a similar variation in the control schools. Therefore, it is difficult to attribute this variation between schools to facilitators alone. Unfortunately, we did not have enough statistical power to test this hypothesis.

We found an interaction between group and baseline BDI-II score. Those students who had mild and moderate depressive symptoms at baseline had a greater likelihood of recovering than those with more severe symptoms in the intervention group but not in the control arm. One potential explanation is that the length and intensity of the intervention (8 sessions) was insufficient for those students with more severe symptoms, something supported by a recent systematic review [20]. We also explored if there was a difference in adherence rate to the sessions according to baseline BDI-II score, hypothesizing that those students with mild to moderate severity of depressive symptoms (<30 points) may have had a higher adherence rate, but adherence to session was fairly similar among all severity levels of depression (results available from authors under request). Notwithstanding the above, all these analyses were merely exploratory as we did not have sufficient statistical power to test adequately any of these hypotheses.

This study has several limitations. Firstly, we could not follow-up nearly 18 % of students, mainly because they changed schools and there was no information of the schools the students moved in. Considering that this dropout was similar in both arms, there is just a small chance that some attrition bias could have been introduced. Secondly, the attendance rate to our intervention was lower than it has been reported from other similar interventions. This may be considered as a failure in delivering a more attractive intervention, but it might also related to the personal circumstances of the students and a rather adverse socio-economic context. Even though this intervention was based on a previous universal intervention, which included an extensive formative work; for our study, we did not conduct a pilot study to assess adolescents’ perceptions and preferences. Finally, we cannot be certain about the long-term effect of the intervention because we only considered a follow-up of 3 months after intervention. Some studies have found that it could take some time for an intervention to show any effects [24].

## Conclusions

We found no clear evidence of the effectiveness of a brief, indicated school-based intervention based on cognitive-behavioural models on reducing depressive symptoms among Chilean adolescents from low-income families. Future studies need to address the limitations we had conducting our trial, and to test interventions that include broader preventive strategies for at-risk adolescents, consider other strategies for emotional and behavioural change [53] and to include mechanisms, within the intervention, that encourage adherence to psychosocial interventions, for example, using new technologies such as social media and/or gaming. Interventions that address a broader range of problems and symptoms might also be worth exploring. Finally, there is room to think of interventions that use multiple delivery forms including peer-to-peer and teacher components.

## Additional file

Additional file 1: Annex: Primary analysis with imputed data: recovery rate at 3 months (This table can be compared to Table 2 in paper). (DOC 226 kb)
