# Supplementary material for: Indicated school-based intervention to improve depressive symptoms among at risk Chilean adolescents: a randomized controlled trial
Source: BMC Psychiatry. 2016 Aug 4;16:276. doi: 10.1186/s12888-016-0985-4 (PMC4973098; doi:10.1186/s12888-016-0985-4)
Supplement: Additional file 1: — Annex: Primary analysis with imputed data: recovery rate at 3 months (This table can be compared to Table 2 in paper). (DOC 226 kb) [file 12888_2016_985_MOESM1_ESM.doc]

**Annex: Primary analysis with imputed data: recovery rate at 3 months (This table can be compared to Table 2 in paper)**

| **Control (N=552)**  **n (% recovery rate)** | **Intervention (N=1087)**  **n (% recovery rate)** | **OR (95% CI)** | **p-value** |
| --- | --- | --- | --- |
|  |  | **Unadjusted** | |
| 267 (48.4) | 550 (50.6) | 1.09 (0.89 to 1.34) | 0.394 |
|  |  | **Adjusted*** | |
|  |  | 1.10 (0.89 to 1.37) | 0.377 |

Note: 63 observations in the recovery variable were imputed 20 times, generating a dataset of 1639 observations. *The adjusted model included sex, age and baseline BDI-II. Recovery rate refers to proportion of participants who scored <10 (among boys) and <15 (among girls) in the BDI-II at 3 months after completing the intervention.
